# A Distinct Clinicopathological Feature and Prognosis of Young Gastric Cancer Patients Aged ≤ 45 Years Old

**DOI:** 10.3389/fonc.2021.674224

**Published:** 2021-08-26

**Authors:** Qian Huang, Xiufeng Zheng, Yang Jiao, Yanna Lei, Xiaoying Li, Feng Bi, Fukun Guo, Gang Wang, Ming Liu

**Affiliations:** ^1^Department of Abdominal Oncology, West China Hospital, Sichuan University, Chengdu, China; ^2^Division of Experimental Hematology and Cancer Biology, Children’s Hospital Medical Center, Cincinnati, OH, United States; ^3^National Engineering Research Center for Biomaterials, Sichuan University, Chengdu, Sichuan, China

**Keywords:** early-onset gastric cancer, clinicopathological features, overall survival, prognosis, treatment

## Abstract

**Purpose:**

The aim of this retrospective study was to probe into clinicopathological features and prognosis of early-onset gastric cancer (EOGC) patients aged ≤ 45 years old.

**Methods:**

This study selected 154 young gastric cancer patients aged ≤ 45 years old and 158 elderly gastric cancer patients aged > 50 years old admitted to West China Hospital of Sichuan University in 2009-2019 as the research object. These patients were further divided into two groups according to whether tumor can be resected radically. The following parameters were analyzed: age, gender, helicobacter pylori (HP) infection status, Her-2 status, pathological type and stage, chemotherapy, tumor differentiation degree, overall survival (OS).

**Results:**

More than 3,000 patients with gastric carcinoma were screened, and 154 young gastric cancer patients aged ≤ 45 years old were identified as EOGC. Among them, the number of female patients in EOGC group was significantly higher than that of males, accounting for 63.6%. In addition, EOGC were associated with diffuse Laur´en type and poorly differentiated tumors. Interestingly, the Kaplan–Meier method showed that the OS of unresectable EOGC group was significantly lower than that of unresectable LOGC group (*P* = 0.0005) and chemotherapy containing paclitaxel tended to be more effective in the young people (*P* = 0.0511). Nevertheless, there was no significant difference in OS between young and elderly patients with gastric cancer in the radical resection group (*P* = 0.3881).

**Conclusion:**

EOGC patients have a worse prognosis than late-onset gastric cancer (LOGC) patients with advanced unresectable gastric cancer. Palliative surgery or chemotherapy containing paclitaxel may improve the OS of unresectable young individuals with gastric cancer. Additional randomized controlled trials are required for guiding clinical practice.

## Introduction

Gastric cancer is the third leading cause of cancer-related deaths worldwide and half of the deaths from gastric cancer occur in China (1, 2). In the past 10 years, the overall incidence of gastric cancer has gradually decreased (3). However, due to the irregular diet and work schedule, the incidence of early-onset gastric cancer (EOGC), diagnosed in young people has significantly increased (4, 5). At present, there is no clear age limit for early-onset gastric cancer. Regardless of whether it is 30, 40 or 50 years old, the incidence of early-onset gastric cancer is increasing year by year (5). EOGC is different from late-onset gastric cancer (LOGC) that is traditionally common in the elderly people aged > 60 years old (6). Compared with elderly patients, the common characteristics of younger patients include female predominance, faster growth and metastatic property of tumor, worse prognosis, and higher levels of resistance to traditional chemotherapy. In addition, pathological tissues of younger patients are more characteristic of poor differentiation, signet-ring cells carcinoma, and Laur´en diffuse type (4, 7, 8). Because the early symptoms of gastric cancer are not obvious, young patients are more likely to ignore these symptoms. Meanwhile, studies demonstrated that younger patients commonly have more aggressive pathological assessment and worse outcome compared with older patients in different cancer (9, 10). Thus, diagnosis and screening of EOGC patients need to be improved (7, 11).

This study aims to explore clinicopathological characteristics and prognosis of patients with early-onset gastric cancer (≤ 45 years old). In this case-control study, 154 young gastric cancer patients aged ≤ 45 years old and 158 random elderly patients aged > 50 years old who were admitted to West China Hospital of Sichuan University from 2009 to 2019 were selected as the research subjects. Moreover, clinicopathological characteristics and prognosis were analyzed in resectable and unresectable young gastric cancer patients, which could be complementary for current clinical guidance.

## Methods

### Patients

**Figure 1:**
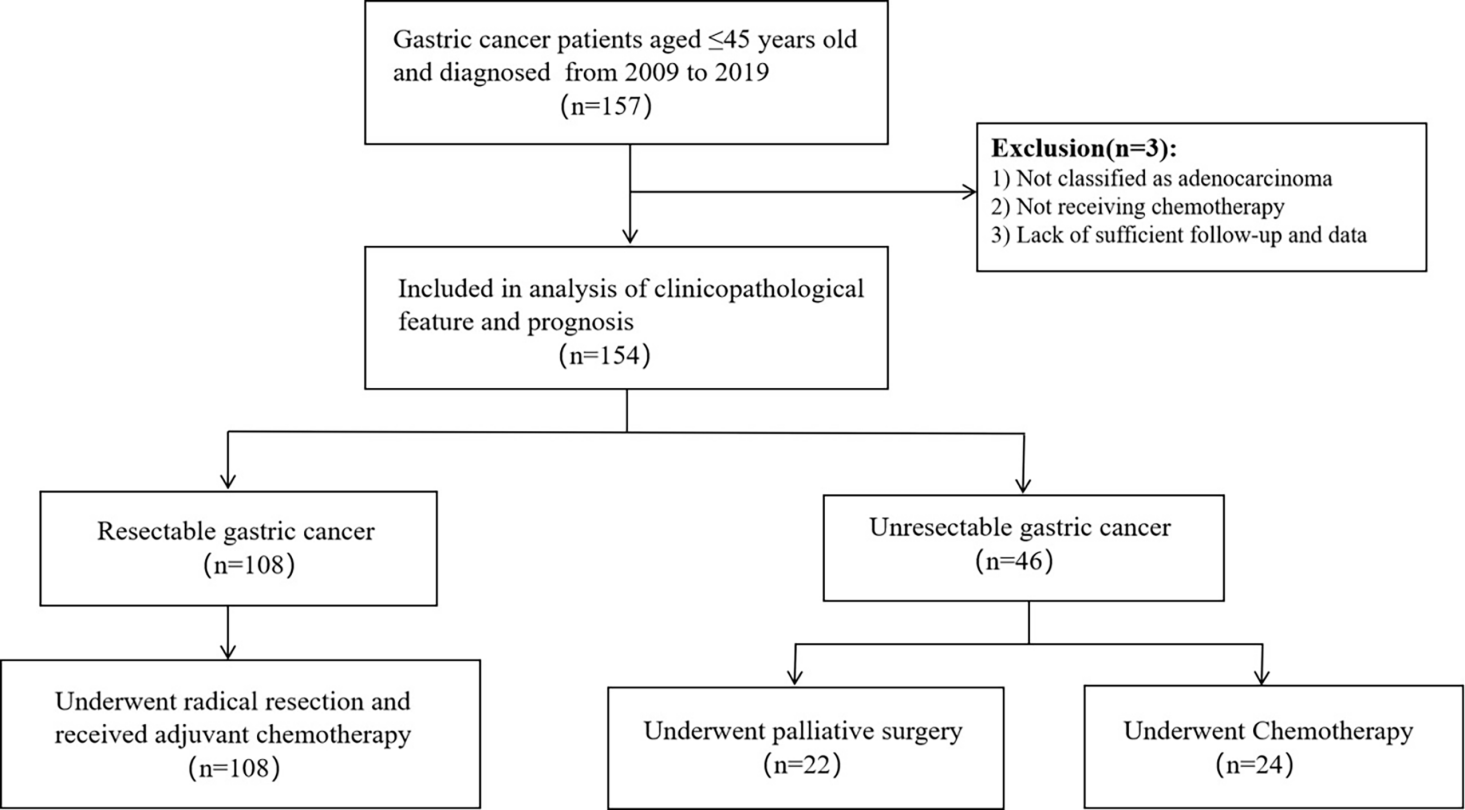
Flowchart of the selection procedure about young gastric cancer.

This was a monocentric, retrospective study. More than 3,000 patients with gastric carcinoma were screened, and 154 young gastric cancer patients aged ≤ 45 years old were identified in West China Hospital of Sichuan University in 2009-2019 as the research subjects. Among them, 108 patients had undergone radical resection and 46 patients were not. We randomly selected 158 patients aged > 50 years old with gastric cancer to serve as a control group, 108 of which had undergone radical surgery and other 50 patients did not. pathological type of gastric cancer in all patients was adenocarcinoma, and all patients received chemotherapy. **Figure 1**, **2** show the selection and matching procedure of the study cohort. At present, there is no clear age criterion for EOGC. According to previous literature and clinical studies, we considered the age of patients ≤ 45 years old as EOGC group, at the same time, those > 50 years old as LOGC group (12, 13).

**Figure 2:**
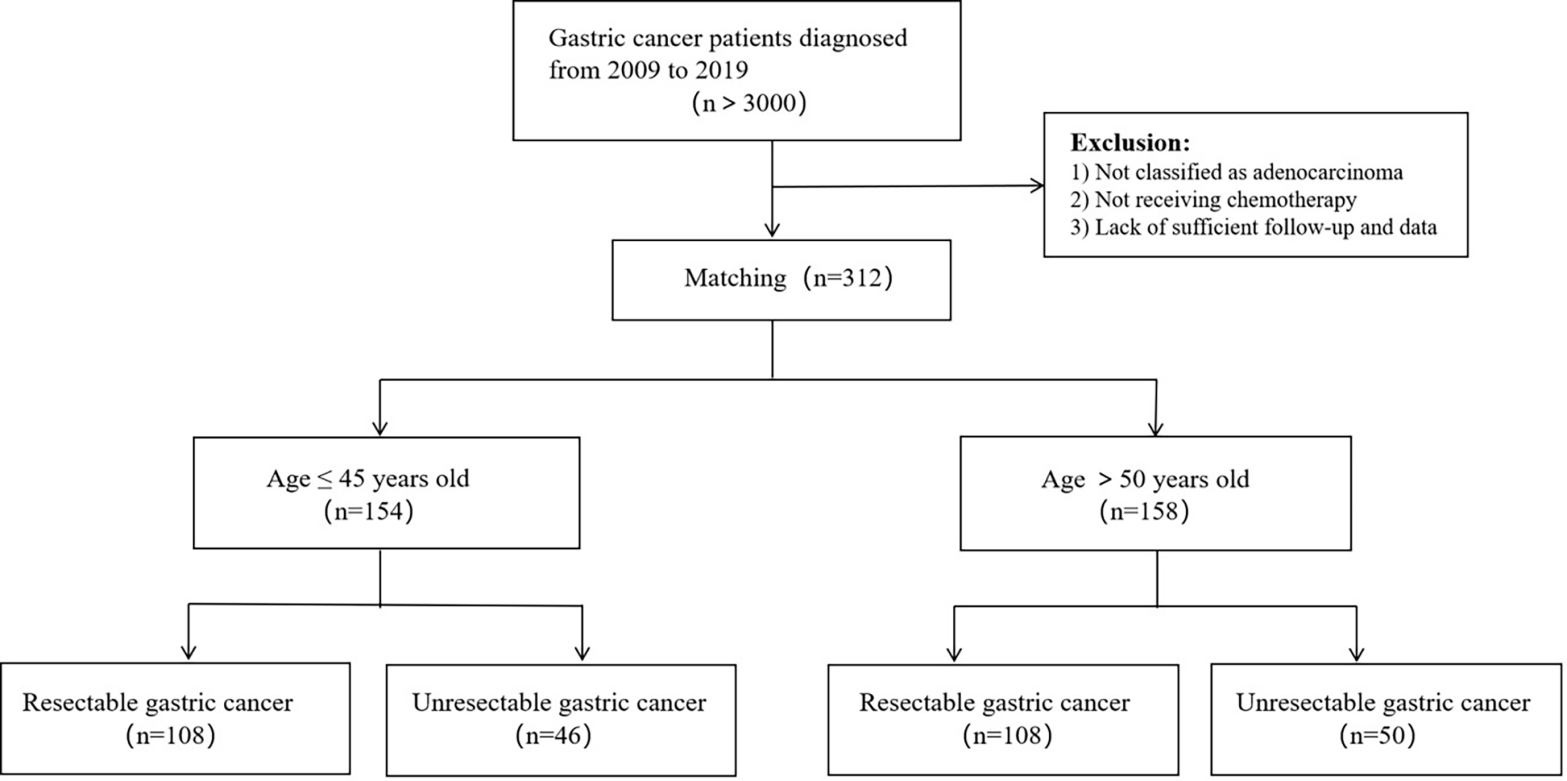
Flowchart of the matching procedure.

### Definitions

Resectable GC was defined as the patients who were absence of distant and implantation metastases and underwent radical gastrectomy with negative cutting edge for stage I-III. Unresectable GC was defined as the advanced tumor invaded large blood vessels and important organs or had distant and/or implantation metastasis (mainly including stage IV and part of stage IIIC). Unresectable gastric cancer also includes locally advanced gastric cancer, although it has undergone radical surgery, the intraoperative or postoperative pathological results suggesting positive margins or implant metastases, etc. Postoperative pathological staging relies on the TNM system designed jointly by the Union for International Cancer Control (UICC) and the American Joint Committee on Cancer (AJCC) in 2017 (8th edition). The degree of differentiation and pathological type of tumor were classified as recommended by the World Health Organization (WHO).

### Data Collection

We reviewed the general information of the patient, surgery status, postoperative pathological results, radiotherapy and chemotherapy status and follow-up. Detailed information of patients including age and sex, location and histological type of tumor, symptom, level of lymph node metastasis, type of distant metastases, stage of disease, operative curability, HP infection and Her-2 status, radiotherapy and chemotherapy were obtained from a retrospective database. Follow-up data included overall survival and pattern of recurrence or metastasis.

### Statistical Analysis

For continuous variables and classified variables, descriptive statistics are expressed as median and absolute numbers and proportions (%), respectively. Group comparison of continuous variables were performed using Student’s t-tests, while categorical variables were compared with Fisher’s exact test or Pearson’s Chi-square test. Correlations between various factors and overall survival of GC were assessed by univariate and multivariate Cox proportional hazards regression analysis. Variables that were deemed of potential importance to the univariate analysis (*P* < 0.100) were included in the multivariate analysis. All *P* values were two sided, and *P* values < 0.050 were considered to be statistically significant. Results for significant prognostic factors were expressed as the hazard ratio for each category and its 95% confidence interval. Patient survival was estimated using the Kaplan–Meier method and log-rank tests were used to evaluate differences in survival among different patient subgroups. The statistical program SPSS version 22.0 (SPSS, Chicago, Illinois, USA) and Graphpad PRISM v. 8.4.3 were used for analysis.

## Results

### Basic Characteristics of Patients With Unresectable Gastric Cancer

We summarized overall clinical and histopathologic features of the patients with unresected gastric cancer in **Table 1**. The median age of 46 patients in EOGC group was 35 years old, among whom five had family history of tumor. EOGC group had a larger proportion of women, while the vast majority are men in LOGC group (*P* < 0.001). Compared with the old-aged cohort, Poor differentiation was significantly more frequent in EOGC group (93.4%; *P* = 0.004). Signet-ring cell carcinoma accounted for 56.5% in EOGC group, while accounted for 28% in LOGC group (*P* = 0.011). Meanwhile, the incidence of peritoneal metastasis was greater in EOGC group(*P* = 0.045). On the contrary, elderly patients are more likely to occur liver metastasis (*P* = 0.027). Paclitaxel-containing chemotherapy was used more frequently in EOGC group than in LOGC group, whereas first-line chemotherapy containing oxaliplatin was used more frequently in the elderly patients (*P* = 0.017). In the history of drinking, young people drank less than old people (*P* = 0.020). No significant differences were found in location of the primary lesion, tumor size, and helicobacter pylori infection status or Her-2 status.

**Table 1:** Patient characteristics with unresectable gastric cancer.

Patient characteristics	EOGC(n = 46,%)	LOGC(n = 50,%)	*P* value
Age			<0.001
(Range)	35 (23-45)	65 (53-81)	
Sex			<0.001
Male	12 (26.1)	38 (76.0)	
Female	34 (73.9)	12 (24.0)	
PS			0.381
0-1	35 (76.1)	34 (68.0)	
2-3	11 (23.9)	16 (32.0)	
Location			0.771
Upper	11 (23.9)	13 (26.0)	
Middle	10 (21.7)	8 (16.0)	
Lower	25 (54.4)	29 (58.0)	
Differentiation			0.004
Poor	43 (93.4)	32 (64.0)	
Moderate	1 (2.2)	6 (12.0)	
Poor-Moderate	1 (2.2)	8 (16.0)	
Unknown	1 (2.2)	4 (8.0)	
Tumor size (cm)			0.625
≤ 5.0	15 (32.6)	14 (28.0)	
> 5.0	31 (67.4)	36 (72.0)	
WHO histological type			0.011
Signet-ring cell carcinoma	26 (56.5)	14 (28.0)	
Tubular adenocarcinoma	0	4 (8.0)	
Both	7 (15.2)	8 (16.0)	
Others or Unknown	13 (28.3)	24 (48.0)	
Palliative surgery			0.683
Yes	22 (47.8)	26 (52.0)	
No	24 (52.2)	24 (48.0)	
Symptom classification			0.553
Epigastric pain	36 (78.3)	36 (72.0)	
Melena/haematemesis	2 (4.3)	3 (6.0)	
Dyspepsia/nausea/vomiting	1 (2.2)	4 (8.0)	
Dysphagia	4 (8.6)	6 (12.0)	
Others	3 (6.6)	1 (2.0)	
HP infection status			0.064
Negative	0	4 (8.0)	
Positive	17 (37.0)	23 (46.0)	
Unknown	29 (63.0)	23 (46.0)	
Alcohol consumption	7 (15.2)	18 (36.0)	0.020
Family history	5 (10.9)	7 (14.0)	0.643
Her-2 status			0.066
Negative or 1^+^	16 (34.8)	26 (52.0)	
2^+^or 3^+^	5 (10.9)	6 (12.0)	
Unknown	25 (54.3)	18 (36.0)	
Celiac lymph node metastases	32(69.6)	37 (74.0)	0.629
Peritoneal metastasis	8 (17.4)	2 (4.0)	0.045
Ovarian metastasis (female)	13 (38.2)	2 (16.7)	0.285
Liver metastasis	6 (13.0)	16 (32.0)	0.027
Bone metastasis	7 (15.2)	2 (4.0)	0.082
First-line chemotherapy			0.017
Containing oxaliplatin	22 (47.8)	38 (76.0)	
Containing paclitaxel	18 (39.1)	9 (18.0)	
Others	6 (13.1)	3 (6.0)	

Values in parentheses are percentages. PS, performance status. HP, helicobacter pylori. Her-2, human epidermal growth factor receptor 2.WHO, world health organization.

### The Features of Patients With Resectable Gastric Cancer

**Table 2** shows the clinical and histopathologic characteristics of patients with resected gastric cancer. The median age of 108 patients in EOGC group was 37(range 27-45) years old, and this group similarly contained a higher proportion of female patients (59.3%) than LOGC group (26.9%) (*P* < 0.001). Poor differentiation was also significantly more frequent in EOGC group (76.9%) than in LOGC group (47.2%) (*P* < 0.001). Meanwhile, WHO histological type in EOGC group contained a larger proportion of signet-ring cell carcinoma (*P* < 0.001) and Laur´en histological type of EOGC group was mainly diffuse-type (*P* < 0.001). Laur´en intestinal-type accounted for only 2.8% in EOGC group but 21.3% in LOGC group. In all patients, the most common symptom was epigastric pain, reported by over 70 percent of patients. Other common presentations were melena/haematemesis, dyspepsia/nausea/vomiting and dysphagia, reported by 1-10 percent of patients. Besides, resectable EOGC group had a lower frequency of Her-2 amplification and overexpression than LOGC group (*P* = 0.001). There was no statistically significant difference in postoperative recurrence and metastasis between the two groups. Although there is a certain difference in pTNM stage, there is no significant differences in T stage and N stage.

**Table 2:** Patient characteristics with resected gastric cancer.

Patient characteristics	EOGC(n = 108,%)	LOGC(n = 108,%)	*P* value
Age			<0.001
(Range)	37 (27–45)	66 (50–79)	
Sex			<0.001
Male	44 (40.7)	79 (73.1)	
Female	64 (59.3)	29 (26.9)	
Location			0.099
Upper	17 (15.7)	9 (8.3)	
Middle	25 (23.1)	19 (17.6)	
Lower	66 (61.2)	80 (74.1)	
Differentiation			<0.001
Poor	83 (76.9)	51 (47.2)	
Poor-Moderate	22 (20.4)	40 (37.0)	
Moderate	1 (0.9)	17 (15.8)	
Unknown	2 (1.8)	0	
Tumor size (cm)			0.359
≤ 5.0	65 (60.2)	66 (61.1)	
> 5.0	22 (20.4)	36 (33.3)	
Unknown	21 (19.4)	6 (5.6)	
WHO histological type			<0.001
Signet-ring cell carcinoma	39 (36.1)	17 (15.7)	
Tubular adenocarcinoma	3 (2.8)	23 (21.3)	
Both	50 (46.3)	32 (29.6)	
Others or Unknown	16 (14.8)	36 (33.4)	
Laur´en histological type			<0.001
Diffuse	65 (60.2)	37 (34.3)	
Mixed	19 (17.6)	13 (12.0)	
Intestinal	3 (2.8)	26 (24.1)	
Unknown	21 (19.4)	32 (29.6)	
Borrmann histological type			0.688
1	1 (0.9)	1 (0.9)	
2	13 (12.1)	19 (17.6)	
3	8 (7.4)	8 (7.4)	
4	9 (8.3)	5 (4.6)	
Unknown	77 (71.3)	75 (69.5)	
pT stage			0.202
T1	19 (17.6)	9 (8.3)	
T2	14 (13.0)	12 (11.1)	
T3	30 (27.8)	35 (32.4)	
T4	45 (41.6)	52 (48.2)	
pN stage			0.073
N0	8 (7.4)	20 (18.5)	
N1	31 (28.7)	24 (22.2)	
N2	22 (20.4)	25 (23.1)	
N3	47 (43.5)	39 (36.2)	
pTNM stage			0.035
IB-IIA	28 (26.0)	20 (18.5)	
IIB-IIIA	23 (21.3)	40 (37.0)	
IIIB-IIIC	57 (52.7)	48 (44.5)	
Adjuvant radiotherapy			0.186
Yes	29 (26.9)	38 (35.2)	
No	79 (73.1)	70 (64.8)	
Symptom classification			0.341
Epigastric pain	85 (78.7)	86 (79.6)	
Melena/haematemesis	11 (10.2)	7 (6.5)	
Dyspepsia/nausea/vomiting	9 (8.3)	6 (5.6)	
Dysphagia	1 (0.9)	2 (1.9)	
Others	2 (1.9)	7 (6.4)	
HP infection status			0.295
Negative	9 (8.3)	6 (5.6)	
Positive	29 (26.9)	39 (36.1)	
Unknown	70 (64.8)	63 (58.3)	
Alcohol consumption	20 (18.5)	48 (44.4)	<0.001
Ovarian metastasis (female)	10 (15.6)	2(6.9)	0.300
Her-2 status			0.001
Negative or 1^+^	75 (69.4)	48 (44.4)	
2^+^ or 3^+^	7 (6.5)	11 (10.2)	
Unknown	26 (24.1)	49 (45.4)	
Recurrence or metastasis(numbers)			0.514
I (pTNM stage)	1 (2.3)	1 (2.1)	
II	5 (11.4)	10 (21.3)	
III-IV	38 (86.3)	36 (76.6)	

Values in parentheses are percentages. TNM, tumor node metastasis; HP, helicobacter pylori; Her-2, human epidermal growth factor receptor 2; WHO, world health organization.

### Univariate and Multivariate Analyses for Overall Survival Associated With Resectability of GC in Young Patients

**Figure 3:**
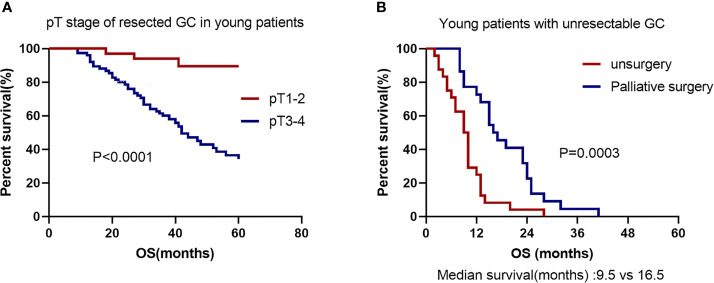
Kaplan–Meier GC survival curve based on significant prognostic predictors for overall survival in EOGC patients with resectability and unresectability. **(A)** pT stage of resectability GC in EOGC patients. **(B)** No surgery or palliative surgery in EOGC patients with unresectable GC.

In this study, curative intent for GC was performed on 108 patients. Among them, 3 patients underwent neoadjuvant chemotherapy before radical operation. IIIB-IIIC stage accounted for 52.7% of pTNM stage in the completely resected EOGC group. Univariate analysis revealed that the poor differentiation, larger tumor size, signet-ring cell carcinoma according to WHO histological type, and higher pT stage, pN stage and pTNM stage all increased death (**Table 3**). In multivariate analyses, only pT stage [hazard ratio (HR) 5.916, 95% CI 1.579-22.173, *P* = 0.008] was the significant prognostic predictor. The OS rate was significantly better in young patients with pT1-2 stage than in those with pT3-4 stage (*P* < 0.0001, **Figure 3A**). All patients received chemotherapy, but simultaneous radiotherapy and chemotherapy did not increase OS compared with chemotherapy alone (*P* = 0.520).

**Table 3:** Univariate and multivariate analyses for overall survival associated with resectability of EOGC patients.

Factors	Univariate analysis		Multivariate analysis	
	HR (95% CI)	*P* value	HR (95% CI)	*P* value
Age	0.915 (0.908,1.024)	0.240		
Sex (female *versus* male)	0.616 (0.328,1.159)	0.133		
Differentiation (poor *versus* others)	2.118 (0.944,4.749)	0.069		
Location (lower *versus* others)	0.842 (0.463,1.531)	0.572		
Tumor size (≤ 5.0 cm *versus* > 5.0 cm)	0.501 (0.249,1.008)	0.053		
WHO histological type (signet-ring cell carcinoma *versus* tubular adenocarcinoma)	0.406 (0.205,0.804)	0.010		
Lauren histological type (diffuse versus others)	0.471 (0.181,1.223)	0.122		
pT stage (T1-2 *versus* T3-4)	0.112 (0.035,0.362)	<0.001	5.916 (1.579,22.173)	0.008
pN stage (N0-1 *versus* N2-3)	0.457 (0.226,0.923)	0.029	0.928 (0.393,2.188)	0.864
pTNM stage (I-II *versus* III-IV)	0.215 (0.091,0.508)	<0.001	1.970 (0.636,6.1.4)	0.240
Adjuvant radiotherapy	1.230 (0.654,2.313)	0.520		
Symptom classification (Epigastric pain *versus* others)	0.879 (0.423,1.826)	0.730		
Her-2 status (≥2^+^ *versus* others)	1.784 (0.427,7.448)	0.428		

Values in parentheses are 95 percent confidence intervals. HR, hazard ratio; CI, confidence interval; WHO, world health organization; Her-2, human epidermal growth factor receptor 2.

### Univariate and Multivariate Analyses for Overall Survival Associated With Unresectability of GC in Young Patients

**Figure 4:**
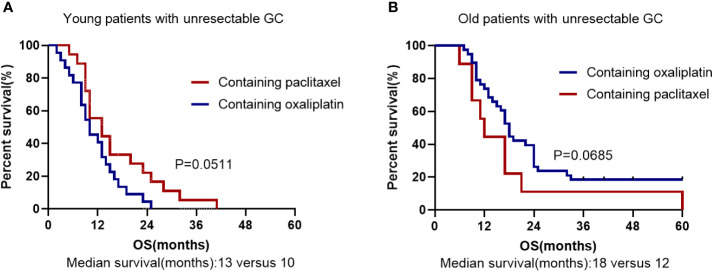
Kaplan–Meier survival curve based on First-line chemotherapy for overall survival in unresected EOGC group **(A)** and LOGC group **(B)**. **(A)** The first-line chemotherapy containing oxaliplatin or paclitaxel in EOGC patients with unresectable GC. **(B)** The first-line chemotherapy containing oxaliplatin or paclitaxel in LOGC patients with unresectable GC.

Unresectability for young GC was performed on 46 patients. Among them, 22 patients (47.8%) underwent surgery with palliative intent and other 24 cases (52.2%) did not undergo surgery. Absence of surgery and cancer family history were considered as significant risk factors for death in the young individuals by univariate analysis (**Table 4**). In multivariate analyses, the palliative surgery [hazard ratio (HR) 0.212, 95% CI 0.088-0.513, *P* = 0.001] was the significant prognostic predictor and first-line chemotherapy with paclitaxel [hazard ratio (HR) 0.490, 95% CI 0.238-1.008, *P* = 0.052] might be a significant prognostic predictor. The OS rate was significantly worse in the patients with no surgery than in those with palliative surgery (*P* = 0.0003; Median survival time 9.5 months *versus* 16.5 months; **Figure 3B**). All patients had undergone chemotherapy. **Figure 4A** shows that the survival rate of EOGC group with paclitaxel in the first-line chemotherapy tended to be better than that with oxaliplatin, although it did not reach statistical significance (Median survival time 13 months *versus* 10 months; *P* = 0.0511). However, in LOGC group, as shown in **Figure 4B**, the survival rate of patients in the first-line chemotherapy with oxaliplatin tended to be better than that with paclitaxel, although there was no statistical significance (Median survival time 18 months *versus* 12 months; *P* = 0.0685).

**Table 4:** Univariate and multivariate analyses for overall survival associated with unresectability of EOGC patients.

Factors	Univariate analysis		Multivariate analysis	
	HR (95% CI)	*P* value	HR (95% CI)	*P* value
Age	1.009 (0.951,1.071)	0.765		
Sex (female *versus* male)	0.608 (0.308,1.197)	0.150		
PS (0-1 *versus* 2-3)	1.184 (0.576,2.435)	0.645		
Location (Lower *versus* others)	0.949 (0.525,1.718)	0.864		
Tumor size (≤ 5.0 cm *versus* > 5.0 cm)	0.758 (0.401,1.434)	0.395		
WHO histological type(signet-ring cell carcinoma *versus* others)	0.711 (0.362,1.399)	0.323		
Palliative surgery (Yes *versus* No)	0.343 (0.182,0.647)	0.001	0.212 (0.088,0.513)	0.001
Symptom classification (Epigastric pain *versus* others)	1.398 (0.667,2.930)	0.375		
Alcohol consumption	1.905 (0.835,4.346)	0.126		
Cancer family history	2.624 (0.991,6.949)	0.052	0.851 (0.295,2.455)	0.765
Her-2 status (≥ 2^+^ *versus* others)	0.432 (0.143,1.304)	0.137		
Celiac lymph node metastases	1.214 (0.634,2.326)	0.558		
Peritoneal metastasis	0.842 (0.454,1.562)	0.586		
Ovarian metastasis(female)	1.243 (0.609,2.536)	0.551		
Liver metastasis	1.083 (0.424,2.767)	0.868		
First-line chemotherapy (oxaliplatin *versus* paclitaxel)	0.516 (0.243,1.093)	0.084	0.490 (0.238,1.008)	0.052

Values in parentheses are 95 percent confidence intervals. HR, hazard ratio; CI, confidence interval; PS, performance status; WHO, world health organization; Her-2, human epidermal growth factor receptor 2.

### Survival Analysis

**Figure 5:**
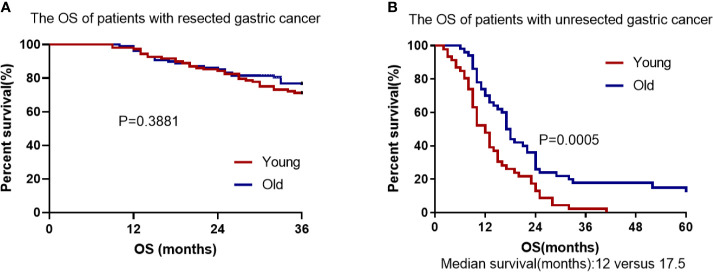
Cumulative survival in EOGC and LOGC patients with resected or unresected GC. **(A)** There was no difference in overall survival between resected EOGC group and LOGC group; *P* = 0.3881 (log-rank test). **(B)** There was statistical significance in overall survival between unresected EOGC group and LOGC group; *P* = 0.0005 (log-rank test).

The total follow-up time of the resectable group was 3 years during which 32 patients died in EOGC group compared with 26 in LOGC group. There was no significant difference in 3-year OS rates between EOGC group and LOGC group after radical operation, which were 70.4% and 75.9%, respectively (*P* = 0.3881, **Figure 5A**). Moreover, 44 patients (40.7%) in EOGC group and 47 patients (43.5%) in LOGC group developed recurrence or metastasis within 3 years (*P* = 0.514; **Table 2**). Among resectable EOGC group, there were 4 patients with gastric recurrence, 10 patients with celiac lymph node metastasis, 6 patients with peritoneal metastasis, 10 female patients with ovarian metastasis, and a number of other patients with rare cases such as liver metastasis, lung metastasis, bone metastasis, and rectal metastasis. It is worthy of note, among them, one patient had very rare breast metastasis.

In the unresectable EOGC group and LOGC group, the median follow-up time was 13.9 months (range 2–41) and 23.1 months (range 6–60), respectively. All patients in unresectable young group died (100%) and 43 patients in unresectable old group died (86%) during follow-up. The OS rate was significantly worse in EOGC group than that in LOGC group (*P* = 0.0005, **Figure 5B**) and the median survival time was 12 months in EOGC group *versus* 17.5 months in LOGC group.

## Discussion

In the younger adults with gastric cancer, the lesions mainly occurred in the fundus and antrum and 87 patients had HP infection (56.5%), similar to that of seniors, more than half of the patients infected. There were 23 cases of ovarian metastases in the female patients (23.5%) and 78 patients died during follow-up (50.6%) in the young adults compared with that 69 old patients died (43.7%). As in previous studies, we found that the young patients were mainly female, and most of them were diagnosed with advanced stage (7, 14). The young patients usually exhibit diffuse type and are likely to metastasize to peritoneal (15, 16). Meanwhile, EOGC group was also featured with higher proportion of poor differentiation and signet-ring cell carcinoma, suggesting that EOGC may be more aggressive (17). We further found a higher proportion of peritoneal metastasis in the young patients with advanced gastric cancer and a higher proportion of liver metastasis in the elderly patients. It has been suggested that helicobacter pylori infection is closely related with the occurrence and development of gastric cancer (18). Thus, HP-infection screening and treatment are deemed the most cost-effective strategies to control gastric cancer among young people with high incidence of gastric cancer (19, 20).

In agreement with the previous studies (8, 21), we found no significant difference in OS between the older and younger patients with resected gastric cancer (*P* = 0.3881). However, in the unresected gastric cancer groups, for the first time we found that there was significant difference in OS between the older and younger individuals (*P* = 0.0005). Our findings suggest that the prognosis of young individuals with advanced or unresectable gastric cancer is worse than that of elderly patients and age is a significant independent factor associated with worse prognosis in patients with unresectable gastric cancer. Our study also found that palliative resection can improve the survival of young patients with incurable gastric cancer (*P* = 0.0003) (22, 23). Therefore, palliative resection may be considered for advanced and incurable young patients with good basic physical condition. Furthermore, our study suggests that younger people with advanced unresectable gastric cancer can benefit more from first-line chemotherapy containing paclitaxel than that containing oxaliplatin (*P* = 0.0511) (24). We speculate that the higher proportion of signet-ring cell carcinoma in unresectable young patients account for the better efficacy of paclitaxel-containing chemotherapy, given that paclitaxel-containing chemotherapy is more effective in patients with advanced gastric cancer with peritoneal metastasis or signet-ring cell carcinoma (17, 25, 26). Furthermore, we found that only 12 younger patients had positive Her-2 overexpression (≥ 2^+^), suggesting that resectable EOGC patients has a lower frequency of Her-2 amplification and overexpression than LOGC patients (*P* = 0.001) (27). In addition, our study found that adjuvant radiotherapy combined with chemotherapy did not improve OS compared with chemotherapy alone, which is consistent with previous studies (28).

The incidence of young patients with gastric cancer is less affected by environmental factors and more related with gene mutation (12). Studies have pointed out that first-degree relatives of patients with EOGC increases risk to gastrointestinal cancer (29). Due to the limited research conditions, our study did not sequence the exons of tumor tissues. Nonetheless, based on the previous research, we can draw a conclusion that EOGC has distinct genomic alterations and diffuse histologic features. Germline mutations in CDH1 occur in approximately 40% of families with hereditary diffuse gastric cancer (HDGC). However, studies have found that there are also CDH1 germline mutations in EOGC (30, 31). Integrative genomic analysis found that higher proportions of early-onset diffuse gastric cancers (DGCs) contain somatic mutations in CDH1 which were associated with shorter survival times compared with late-onset DGCs (32–34). Interestingly, no clear CDH1 variants were found in Brazilian EOGC patients, and eating habits may be related to the development of EOGC (35). According to the integrative analysis of mRNA and protein data, EOGC was divided into four subtypes in which Subtype2 and 4 are associated with immunity (long survival) and invasive tumors (short survival), respectively (36). ARID1A is one of the most frequently mutated genes in gastric cancer. A study found that high heterogeneity of ARID1A expression was associated with increased tumor infiltrating lymphocytes (TILs) density in EOGC (37). Therefore, the abrupt landscape of EOGC and LOGC is very different.

It is of note that, our study has some limitations. First, this was a single-center retrospective analysis and thus it is impossible to assess all potential confounding factors. Secondly, due to the large time span, this analysis could not accurately reflect the current clinical practice of gastric cancer. Thirdly, our cohort did not include data of genetic information, which may ignore the role of age-specific molecular biological characteristics in the prognosis of young patients with gastric cancer. Further analysis of internal biological characteristics is needed in combination with second-generation sequencing or full-exon sequencing. Lastly, the sample size was small, and the drug sensitivity of young patients with gastric cancer needs to be further confirmed by prospective large clinical data.

## Conclusions

The clinicopathological features of young patients with gastric cancer included: female predominance, poor differentiation, large proportion of signet-ring cell carcinoma, advanced stage at diagnosis, and likelihood to metastasize to peritoneal. There was no difference in OS between young patients and old patients in resectable group. However, in unresectable group, the prognosis of young patients was obviously worse than that of elderly patients. In terms of treatment, compared with traditional first-line chemotherapy including oxaliplatin, Paclitaxel-containing chemotherapy had greater benefits for unresectable young patients.

## Data Availability Statement

The original contributions presented in the study are included in the article/supplementary material. Further inquiries can be directed to the corresponding author.

## Ethics Statement

This retrospective chart review study involving human participants was in accordance with the ethical standards of the institutional and national research committee and with the 1964 Helsinki Declaration and its later amendments or comparable ethical standards. The Human Investigation Committee (IRB) of Sichuan University approved this study.

## Author Contributions

All authors contributed to the article and approved the submitted version. Material preparation, data collection and analysis were performed by QH, YJ, YL, XL, XZ, FB, GW, FG, and ML. The first draft of the manuscript was written by QH.

## Funding

This work was supported by Sichuan Science and Technology Program (Grant No.2020JDRC0025) and 1.3.5 Project for Disciplines of Excellence, West China Hospital, Sichuan University (Grant No. ZYJC21043).

## Conflict of Interest

The authors declare that the research was conducted in the absence of any commercial or financial relationships that could be construed as a potential conflict of interest.

## Publisher’s Note

All claims expressed in this article are solely those of the authors and do not necessarily represent those of their affiliated organizations, or those of the publisher, the editors and the reviewers. Any product that may be evaluated in this article, or claim that may be made by its manufacturer, is not guaranteed or endorsed by the publisher.
